# Changes in the Concentration of Markers Participating in the Regulation of the Apoptosis Receptor Pathway Involving Soluble Tumour Necrosis Factor Ligand Inducing Apoptosis (sTRAIL) and Osteoprotegerin (OPG) in the Serum of Women with Ovarian Cancer—Participation in Pathogenesis or a Possible Clinical Use?

**DOI:** 10.3390/cells9030612

**Published:** 2020-03-04

**Authors:** Aleksandra Mielczarek-Palacz, Zdzisława Kondera-Anasz, Marta Smycz-Kubańska

**Affiliations:** Department of Immunology and Serology, Faculty of Pharmaceutical Sciences in Sosnowiec, Medical University of Silesia, 40-055 Katowice, Poland; zanasz@sum.edu.pl (Z.K.-A.); mkubanska@sum.edu.pl (M.S.-K.)

**Keywords:** osteoprotegerin, TRAIL, ovarian cancer, apoptosis

## Abstract

Due to the ability to selectively induce apoptosis in cancer cells, the most interesting target for clinical research is the tumour necrosis factor ligand inducing apoptosis (TRAIL), which binds specific receptors, including osteoprotegerin (OPG). The aim of the study was to analyse the concentration of soluble TRAIL (sTRAIL) and OPG in the serum of women with serous or mucinous ovarian cancer, taking into account different levels of cancer histological differentiation. The group included 97 women with the diagnosed *Cystadenocarcinoma papillare serosum IIIc* and *Cystadenocarcinoma mucinosum IIIc*. Concentrations of parameters were measured by ELISA. Analysis of the obtained results showed a statistically significantly higher concentration of sTRAIL and OPG in the serum of women with ovarian serous and mucinous cancer compared to the control group (*p* < 0.0001). Statistical significance was found between sTRAIL and OPG concentration in G1 and G3 serous cancer (*p* < 0.01) and in OPG mucinous cancer between G1 and G3 (*p* < 0.01) and G2 and G3 (*p* < 0.01). An important role in the pathogenesis of ovarian cancer is played by disorders of the apoptosis process involving the sTRAIL/OPG system, which are associated with the histological type and the degree of histological differentiation of the tumour. Determining the concentration of tested parameters in combination with other markers may be useful in the future in the diagnosis of ovarian cancer, but that requires further research.

## 1. Introduction

Ovarian cancer is still one of the worst-prognosis gynaecological cancers with the highest mortality in most countries in the world, and unfortunately, statistical incidence studies predict that by 2040 mortality will increase significantly [1,2]. It is caused by asymptomatic tumour growth, lack of characteristic clinical symptoms at the beginning of the disease, and a lack of diagnostic tests helpful in early diagnosis [3]. For this reason, research is still underway that focuses on seeking new prognostic and diagnostic markers and assessing their possible usefulness in clinical practice [4].

Literature shows that in the formation and development of ovarian tumours, an important role is played by disorders of the apoptosis process, which may be regulated by soluble ligands and their receptors belonging to the superfamily of tumour necrosis factor molecules (TNF), and the ability to monitor them by measuring them in blood serum might find possible clinical use.

Studies conducted so far have shown that ovarian tumours are accompanied by changes in the concentration of markers monitoring the process of apoptosis in the following ligand/receptor systems: TNF/sTNF-R, sCD30/sCD30L, sCD40/sCD40L, and soluble tumour necrosis factor ligand inducing apoptosis (sTRAIL)/sTRAIL-R1 and R2. These changes indicate disturbances in the extrinsic pathway of apoptosis induction and may constitute one of the immune mechanisms supporting the development of ovarian cancer. In addition, sTNF-R1, sTNF-R2, sCD30, sCD30L, and sCD40 concentrations have been shown to be useful in the early diagnosis of ovarian cancer, and sCD40L, sTRAIL-R1, and sTRAIL-R2 in the differential diagnosis of benign tumours [5,6,7].

Among the molecules belonging to the apoptosis receptor pathway, the most interesting target of clinical research is the tumour necrosis factor ligand that induces TRAIL (TNF-related apoptosis inducing ligand) [8,9,10,11]. TRAIL is a potent stimulator of apoptosis in cancer cells and an important immune effector molecule in the surveillance and elimination of developing tumours [12].

As shown by the studies conducted so far, TRAIL may also show nonapoptotic functions supporting tumour development in cancer cells resistant to apoptosis [13,14]. TRAIL could induce metastasis and activate cancer survival pathways [15,16]. Von Karstedt et al. [17] showed that tumour cell-expressed TRAIL and TRAIL-R2 promote cancer progression, invasion, and metastasis by cancer cell-autonomous activation of Rac1 via the membrane-proximal domain (MPD) of TRAIL-R2 independently of fas-associated death domain (FADD), and according to Hartwig et al. [18] TRAIL is the main immune mechanism supporting cancer growth in colorectal and pancreatic cancer lines, which may also apply to other types of cancers.

There are five types of TRAIL receptors. They are four membrane receptors TRAIL-R1 (DR4), TRAIL-R2 (DR5), TRAIL-R3 (DcR1), and TRAIL-R4 (DcR1), and one soluble receptor called osteoprotegerin (OPG) [19,20]. Death receptors (DR) have the ability to induce apoptosis through a combination with TRAIL, creating TRAIL-R1 (DR4) and TRAIL-R2 (DR5) [21,22]. In contrast, the other receptors are ‘trap’ receptors (DcR decoy receptors) that do not induce apoptosis by competing with DR4 and DR5 for ligand attachment. These decoy receptors act as inhibitors of the TRAIL-induced tumour suppression and are involved in tumoral resistance [23].

Due to the demonstrated anti-apoptotic effect of osteoprotegerin in patients with ovarian cancer [24], and the fact of the presence of this protein in soluble form, it seems interesting to know also the systemic changes occurring in the sTRAIL/OPG system and to assess the possible diagnostic usefulness of the determination of the concentration of tested parameters in female serum with ovarian cancer.

Therefore, the aim of the study was to analyse the concentration of soluble tumour necrosis factor ligand inducing apoptosis (sTRAIL) and its osteoeprotegerine receptor (OPG) in the serum of women with serous or mucinous ovarian cancer, taking into account the histological differentiation of the cancer.

## 2. Material and Methods

The study group included 97 women, aged 19 to 78 (mean age: 55.32 ± 14.61 years) with the diagnosed ovarian cancer. The group included 52 women with the diagnosed *Cystadenocarcinoma papillare serosum III c* (14 had G1, 18 had G2, and 20 had G3 staging) and 45 women with the diagnosed *Cystadenocarcinoma mucinosum III c* (12 had G1, 15 had G2, and 18 had G3 staging). Staging employed the criteria recommended by the International Federation of Gynaecology and Obstetrics (FIGO). The assessment of the degree of histological differentiation of cancer (grading) is presented according to the following scale: G1—highly differentiated, G2—moderately differentiated, G3—poorly differentiated. The diagnosis of tumours was done on the basis of clinical symptoms, results of gynaecological and histopathological examination, and laboratory tests. The women qualified to the studied group were clinically diagnosed with ovarian tumour confirmed with a histopathological examination. Other coexisting disorders of the reproductive organs were excluded. Research material came from the Clinical Ward of Obstetrics and Gynecology, Medical University of Silesia. In addition, 42 healthy women aged between 28–67 (mean age: 47.74 ± 10.64 years) were included in the control group. These women did not have any gynaecological diseases.In the study group, blood was taken from the women before surgery. Blood was collected from these women to obtain serum, and then centrifuged at 1500× *g* for 15 min and stored for testing at −80 °C in small portions.

However, blood in women from the control group was collected while they reported for control tests. The collected biological material was handled in the same way as for the study group.

Quantitative immunoenzymatic ELISA was performed in both the test and control groups using the following tests: TRAIL/Apo2L Diaclone and MicroVue OPG EIA Kit from Qudiel Corporation. Tests sensitivity amounted to 64 pg/mL and 0.04 pmol/L. Tumour CA 125 (cancer antigen-125) antigen concentration was determined by microparticulate immunoenzymatic method (MEIA) using Abbott Diagnostics Tumour Markers CA-125 ^TM^ MEIA kits, test sensitivity was 2 U/mL.

The conduct of these studies was approved by the ethics committee of the Medical University of Silesia in Katowice and all women participating in the study.

The obtained test results were subjected to statistical analysis using the Statistica 13.3 program. The normality of the distribution of tested variables was checked using the Shapiro–Wilk test. For the parameters tested, the median and quartile range were determined, and the results obtained were compared using the Mann–Whitney test.

The Spearman’s rank test was used to perform the correlation, giving the correlation coefficient (r), and the statistically significant level was assumed to be *p* < 0.05.

## 3. Results

In the serum of healthy women belonging to the control group and women with ovarian cancer, sTRAIL concentration was determined. Due to the fact that the values obtained did not correspond to the normal distribution, the results were presented in the form of median and lower and upper interquartile range (Q1 and Q3). In the group of healthy women, Q1 and Q3 were, respectively, 490.00 and 822.67, while the median value was 640.00 pg/mL. In the group of women with ovarian cancer, Q1 and Q3 were, respectively, 2001.00 and 3700.00, with the median equal to 2733.00 pg/mL. A statistically significantly higher concentration of the studied parameter in the serum of women with ovarian cancer was found compared to that in the control group (*p* < 0.0001). Next, the serum sTRAIL concentration in women with serous and mucinous ovarian cancer was analysed. In the serum of women with serous ovarian cancer Q1 and Q3 were, respectively, 2213.50 and 3839.00, and the median value was 3020.50 pg/mL, while in the group of women with mucinous ovarian cancer, Q1 and Q3 were 1711.00 and 3200.00, with a median of 2450.80 pg/mL. Statistically significantly higher serum sTRAIL concentration was found in both women with serous and mucinous histological types of cancer compared to those in the control group (*p <* 0.0001). In addition, the analysis of the research results showed statistical significance between the concentration of the studied parameter depending on the degree of histological differentiation of G1 (highly differentiated) and G3 (poorly differentiated) serous and mucous carcinomas (*p* < 0.01). However, no significant differences were found between the serum concentration of sTRAIL in women with serous and mucinous ovarian carcinoma moderately differentiated (G2) and poorly differentiated (G3).

**Figure 1:**
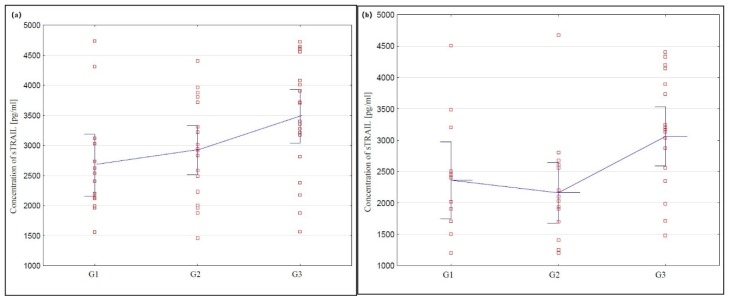
(**a**) Concentrations of soluble tumour necrosis factor ligand inducing apoptosis (sTRAIL) in serum of women with serous ovarian cancer depending on the degree of differentiation G1 (highly differentiated) G2 (moderately differentiated), and G3 (poorly differentiated). (**b**) Concentrations of sTRAIL in serum on women with mucinous ovarian cancer depending on the degree of differentiation G1, G2 and G3.

The obtained results are presented in Figure 1a,b.

In the next stage of the statistical analysis, a correlation test was performed, which showed the existence of a relationship between the TRAIL concentration and the CA 125 marker concentration in the blood serum of the examined women. A positive (r = 0.63) statistically significant (*p* < 0.0001) correlation was found between variables.

**Figure 2:**
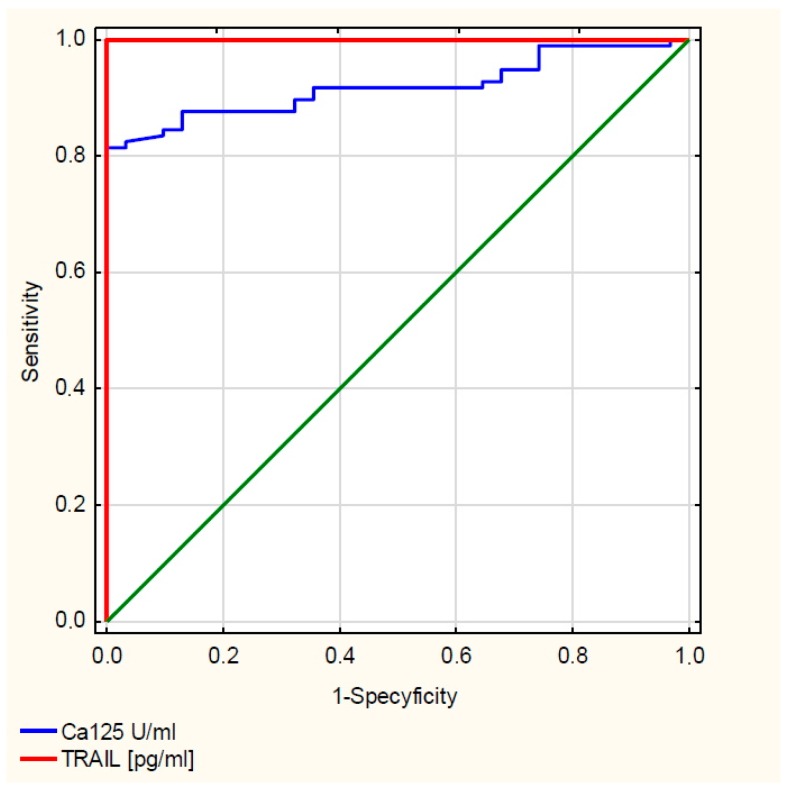
Receiver operating characteristic (ROC) TRAIL curve and CA 125 antigen.

Then, for TRAIL and the CA 125 antigen, the ROC (receiver operating characteristic) curves were drawn, which are illustrated in Figure 2, and the values of the AUC (area under curve) areas were determined, for TRAIL AUC = 1, and for CA125 = 0.919.

The diagnostic value of TRAIL and CA 125 antigen was assessed by determining the sensitivity, specificity, positive predictive value (PPV) and negative predictive value (NPV) for these parameters, as shown in Table 1. For CA 125 antigen the cut-off point was taken to be 35 U/mL, while for TRAIL the cut-off point was 1200 pg/mL.

**Figure 3:**
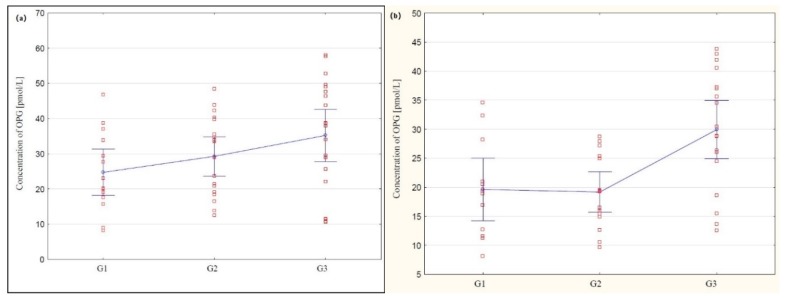
(**a**) Concentrations of osteoprotegerin (OPG) in serum on women with serous ovarian cancer depending on the degree of differentiation G1, G2, and G3. (**b**) Concentrations of OPG in serum of women with mucinous ovarian cancer depending on the degree of differentiation G1, G2, and G3.

Further analysis included assessment of osteoprotegerin (OPG) serum concentration in healthy women in the control group and women with ovarian cancer. The obtained values did not correspond to the normal distribution, therefore, the results were presented in the form of median and lower and upper interquartile range (Q1 and Q3). In the group of healthy women, Q1 and Q3 were, respectively, 2.76 and 8.70, while the median value was 4.70 pmol/L, and in the group of women with ovarian cancer, Q1 and Q3 were 16.90 and 37.00, with a median equal to 26.00 pmol/L. A statistically significantly higher concentration of the studied parameter in the serum of women with ovarian cancer was found compared to the concentration in women from the control group (*p* < 0.0001). Next, the OPG concentration in the serum of the examined women was analysed depending on the histological type of ovarian cancer. In the serum of women with serous ovarian cancer, Q1 and Q3 were, respectively, 19.01 and 40.00, and the median value was 29.45 pmol/L. In the group of women with mucinous ovarian cancer, Q1 and Q3 were 15.46 and 28.90, with a median of 21.00 pmol/L. Statistically significantly higher serum OPG concentration was found in both women with serous and mucinous histological types of cancer compared to those in the control group (*p* < 0.0001). In addition, the analysis showed statistical significance between OPG concentration depending on the degree of histological differentiation in women with serous ovarian cancer between G1 and G3 (*p* < 0.01), and in women with mucinous cancer between G1 and G3 (*p* < 0.01) and G2 and G3 (*p* < 0.01). The obtained results are shown in Figure 3a,b.

**Figure 4:**
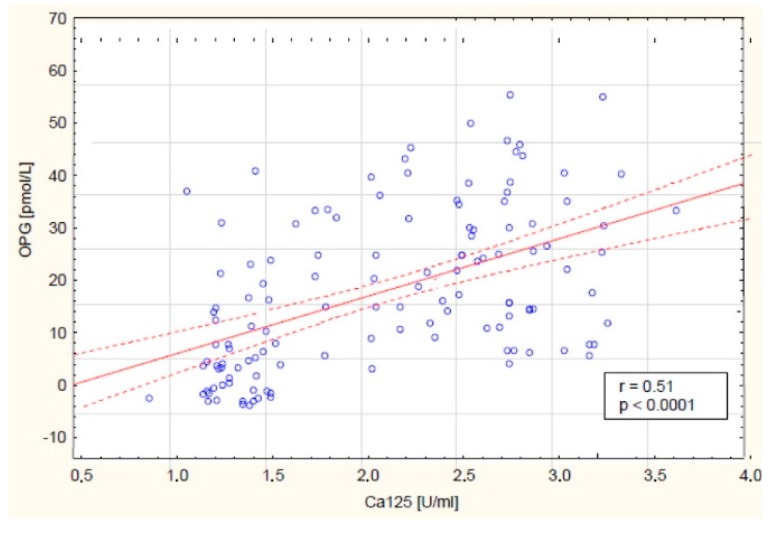
Linear regression curve showing the relationship between OPG concentration and the concentration of CA 125 antigen in the blood serum of women with ovarian cancer.

Further analysis showed the relationship between the OPG concentration and the CA 125 marker concentration in the blood of the examined women. A positive (r = 0.51) and statistically significant (*p* < 0.0001) correlation was found between the variables. The linear regression curve showing this relationship is presented in Figure 4.

**Figure 5:**
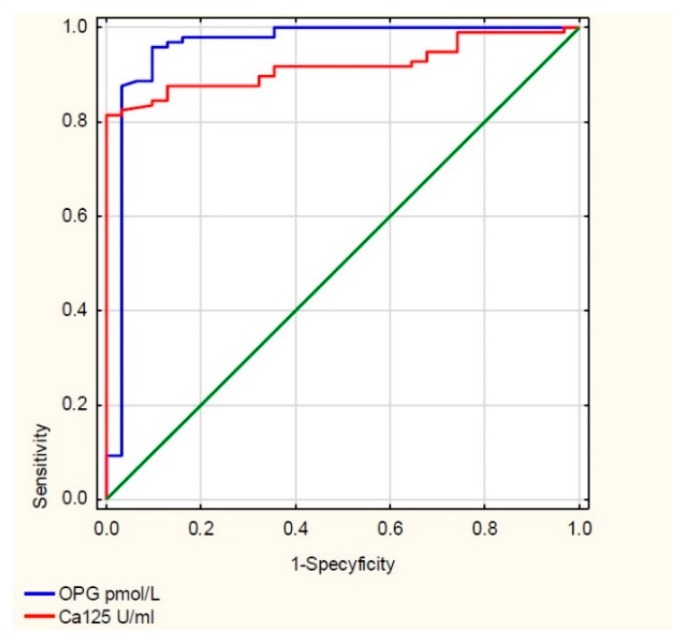
ROC OPG curve and CA 125 antigen.

Then, for OPG and the CA 125 antigen, ROC curves were drawn, illustrated in Figure 5, and the values of the areas under them, the AUCs, were determined. For OPG, AUC = 0.957, and for CA125, AUC = 0.919.

The diagnostic value of OPG and CA 125 antigen determination in the serum of the examined women was also assessed by determining the sensitivity, specificity, positive predictive value, and negative predictive value for these parameters, as shown in Table 2. For CA 125 antigen, the cut-off point was 35 U/mL, while for OPG the cut-off point was 10.5 pmol/L.

## 4. Discussion

Ovarian cancer is the largest cause of death among gynaecological cancers. This disease is initially asymptomatic and, therefore, is usually detected in advanced stages when complete recovery is most often impossible [25]. Research on the biology of this cancer has been ongoing for many years and there are still no effective diagnostic markers with adequate sensitivity and specificity that would be useful in early diagnosis. Recent years have brought a new look at the origin and histogenesis of this cancer. For this reason, new research directions have emerged aimed not only at seeking diagnostic and prognostic markers, but also methods of targeted therapy aimed at blocking tumour-specific molecular pathways responsible for its development and expansion.

Literature shows that apoptosis disorders play an important role in the pathogenesis of ovarian tumours, which may be regulated by soluble ligands and their receptors belonging to the superfamily of TNF tumour necrosis factor molecules. These changes might be associated with systemic and local immunosuppression associated with increased levels of soluble molecules appearing in body fluids, including serum.

A typical ligand of the receptor pathway involved in the selective induction of cancer cell apoptosis is TRAIL, which works by binding specific surface receptors located on target cells—TRAIL-R1 (DR4) and TRAIL-R2 (DR5). Receptors may also exist in soluble forms. Studies in the serum of women with ovarian cancer have shown an increased serum concentration of sTRAIL in women with ovarian serous cancer and a benign tumour—serous adenocarcinoma and mature teratoma of the ovary. It has also been demonstrated that the determination of sTRAIL-R1 and sTRAIL-R2 may prove useful in the differential diagnosis of benign ovarian tumours. According to the authors, changes in the concentration of the studied parameters testified to apoptosis disorders in women with ovarian cancer involving the sTRAIL/sTRAIL-R1 and sTRAIL/sTRAIL-R2 systems [7].

The promising results obtained induced us to further analysis, which included assessing the serum concentration of sTRAIL ligands in women diagnosed with different types of cancer, including serous (*Cystadenocarcinoma papillare serosum IIIc* according to FIGO) and mucinous (*Cystadenocarcinoma mucinosum IIIc* according to FIGO). A significantly increased sTRAIL concentration in the serum of women with both serous and mucinous cancer compared to the control group was demonstrated, which indicates the disturbance of the extrinsic apoptosis process dependent on the histological type of ovarian cancer, which might be one of the immunological mechanisms involved in pathogenesis of these cancers. Interesting observations were also provided by the analysis of sTRAIL concentration, taking into account the degree of histological differentiation of cancer. The demonstrated statistical significance between the concentration of the studied parameter in the serum of women with serous and highly differentiated G1 and poorly differentiated G3 mucinous carcinomas shows that apoptosis disorders involving sTRAIL are also associated with the degree of histological differentiation of ovarian cancer, which could be useful in developing new methods of targeted ovarian cancer therapy. In addition, the analysis showed that there is a correlation between the concentration of sTRAIL and the decimal logarithm of the concentration of CA 125 antigen in the blood serum of women from the study group, which was statistically significant (r = 0.63, *p* < 0.0001), and the assessment of the diagnostic value of the tests showed that the determination of the concentration of sTRAIL and CA 125 in the blood serum is characterised by high sensitivity for sTRAIL and the CA 125 antigen, and the simultaneous determination of the concentration of these parameters in the serum of women may be useful in the diagnosis of ovarian cancer, but requires further research.

Studies on the kinetic assessment of changes in TRAIL as a potential predictive and prognostic factor in the serum of patients with stage IIIC and IV epithelial carcinoma (EOC), according to FIGO, or primary peritoneal carcinoma (PPC) eligible for an interval debulking surgery (IDS) were conducted by Gasowska-Bodnar et al. [26]. The obtained results showed that TRAIL concentration did not differ significantly in patients with ovarian cancer compared to the control group. However, the increase in the concentration of the studied parameter was demonstrated after two cycles of neoadjuvant chemotherapy. According to the authors, TRAIL concentration is not critical as a predictive or prognostic factor in patients with ovarian cancer or primary peritoneal carcinoma [26].

Research conducted so far has shown that TRAIL might participate in both the selective induction of apoptosis in some cancer cells and may perform nonapoptotic functions, including participation in the development of certain cancers and metastasis. For this reason, it still remains the subject of intensive research for possible clinical use.

Literature shows that TRAIL is a promising therapeutic tool in cancerous diseases, including ovarian cancer [27,28,29,30]. Such studies were conducted by Tomek et al. [31], who evaluated in six ovarian cancer cell lines the possible ability of TRAIL ligand using cytotoxic drugs to induce apoptosis. As the result of the conducted research, they found that TRAIL showed significant ability to induce apoptosis in the following ovarian cancer cell lines: MZ-26, CaOV-3, ES-2.

The research showed that TRAIL had a synergistic effect, leading to increased apoptosis after incubation of TRAIL-resistant cell lines with cytotoxic mediators.

According to the authors, the demonstrated ability of TRAIL to induce apoptosis in ovarian cancer cells, as well as to increase the activity of chemotherapeutic agents even in cell lines resistant to TRAIL-induced cytotoxicity, may be of great importance in the fight against this disease [31].

Also, Cuello et al. [32] assessed the effect of the TRAIL molecule on the chemo-resistance of ovarian cancer tumour cells. In the 12 cell lines tested, they showed resistance to chemotherapy and that almost all ovarian cancer cells that are resistant to chemotherapy are also resistant to TRAIL. After ligand administration, tumour growth stopped, which has a significant impact on the treatment of cancerous lesions. According to the authors, the combination of TRAIL and chemotherapy may be useful as a treatment for chemically resistant ovarian cancers [32]. Similar studies were conducted by Abdollahi [33], who assessed the role of TRAIL as a potential factor applicable in the treatment of ovarian cancer. Studies have shown that the presence of IL-8 regulated the expression of TRAIL receptors on the cell surface in ovarian cancer cell lines in vitro. According to the author, the TRAIL-induced apoptosis of ovarian cancer cells plays an important role in the protein kinase pathway activated by the p38 mitogen (MAPK) [33].

Numerous studies are still underway to understand the mechanisms underlying TRAIL resistance in ovarian cancer, including combination therapy with cytotoxic chemotherapy, retinoids, proteasome inhibitors, demethylating agents, Akt inhibitors, and EGFR inhibitors. Many of them have already demonstrated efficacy in restoring TRAIL sensitivity in preclinical studies, and developing new strategies might in the future improve the effectiveness of existing therapies [34].

Understanding these mechanisms also involves TRAIL-specific receptors, including osteoprotegerin, and an assessment of their possible diagnostic and therapeutic use. Osteoprotegerin not only inhibits apoptosis, but it might also induce proliferation by binding to various cell surface receptors, including canonical cell survival and proliferative pathways. OPG also participates in the induction of angiogenesis, facilitating tumour growth [35].

Cross et al. [36] were the first to conduct a study in which they evaluated the proangiogenic effect of OPG in vitro, as well as the correlation of OPG expression by tumour endothelial cells with clinical-pathological data. Their research demonstrated the ability of OPG to support endothelial cell survival, as well as the formation of core-like structures in vitro using an array tubule formation test. In this study, we showed the capability of OPG to support endothelial cell survival and the formation of core-like structures in vitro with the use of a Matrigel tubule formation assay.

According to the authors, the results obtained indicate a significant contribution to OPG expression of endothelium between malignant tumours and benign tissues, and confirm the potential biological role of this molecule in the development and/or maintenance of the vascular system of the tumour.

A clear separation in endothelial OPG expression between malignant tumours and nonmalignant tissues is presented in our data, which confirms the potential biological role of the molecule in the development and/or maintenance of the vascular system of the tumour [36].

In contrast, Lane et al. [37] assessed whether exogenous OPG could provide protection against TRAIL-induced apoptosis, regardless of its ability to act as a decoy receptor for TRAIL. Their research showed that the integrin pathway αvβ3 and αvβ5/FAK/Akt is involved in OPG-induced attenuation of TRAIL-induced apoptosis in ovarian cancer cells. In addition, this study provides new information on the mechanisms by which OPG reduces TRAIL-induced apoptosis, demonstrating that OPG also acts in a manner independent of TRAIL binding [37].

Literature shows that determination of OPG concentration together with other parameters may also be useful in the diagnosis and monitoring of therapy for some cancers, including ovarian cancer. However, no analysis of the OPG concentration in the serum of women with ovarian cancer has been conducted so far, taking into account the histological type of cancer and the degree of histological differentiation of cancer. Such studies were conducted as a part of this work. The analysis showed a significantly increased concentration of OPG in the serum of women with both serous and mucinous cancer compared to the concentration in the control group, which indicates disturbances in the process of extrinsic apoptosis occurring with the participation of OPG, depending on the histological type of ovarian cancer, and may also indicate the autocrine secretion of this receptor by cancer cells. Interesting observations were also provided by the analysis of OPG concentration taking into account the degree of histological differentiation of cancer, which showed statistical significance between the degree of G1 and G3 differentiation in serous cancer, and in mucinous cancer between G1 and G3 as well as G2 and G3, which might be used in the creation of new therapeutic strategies. In addition, it has been shown that there is a positive significant (r = 0.51, *p* < 0.0001) correlation between the OPG concentration and the decimal logarithm of the CA 125 antigen concentration in the blood serum of the test group of women, and the assessment of the diagnostic value of tests showed that the determination of the concentration of OPG and the marker CA 125 in blood serum is characterised by high sensitivity for OPG and the CA 125 antigen, which indicates that the simultaneous determination of the concentration of these parameters in women’s serum may be useful in the diagnosis of ovarian tumours, but that requires further research.

Similar studies, but in a different biological material, were conducted by Chudecka-Głaz et al. [38], who evaluated the concentration of selected cytokines, proteins, and growth, including osteoprotegerin in the peritoneal fluid of patients with ovarian cancer or benign gynaecological conditions. The obtained results showed that among the parameters examined, only IL-6, IL-8, growth differentiation factor-15 (GDF-15), osteopontin (OPN), and osteonectin were characterised by significant differences in the concentration of peritoneal fluid in patients with ovarian cancer compared with the concentration in patients with other gynaecological disorders. The highest diagnostic value was determined by the concentration of IL-6, GDF-15, and osteonectin. According to the authors, further research on the analysis of selected parameters in peritoneal fluid should be associated with the analysis of GDF-15, stem cell factor (SCF), and OPG, which may contribute to the development of newer, more effective diagnostic and therapeutic methods [38]. Jiang et al. [39] evaluated the expression levels of 174 proteins, including osteoprotegerin, in patients with ovarian cancer using antibody matrix technology and CA125 concentration by ELISA. To conduct the research, they used three discriminatory methods: an artificial neural network, a classification tree, and point analysis at partition points. The analysis showed a panel of five serum protein markers (MSP-alpha, TIMP-4, PDGF-R alpha, OPG, and CA125) that might effectively detect ovarian cancer with high specificity (95%) and high sensitivity (100%), with AUC = 0.98, while CA125 had AUC = 0.87. According to the authors, this is a promising set of serum markers for the detection of ovarian cancer, which may be useful in the diagnosis of this disease [39].

Interesting observations are also provided by studies on the assessment of the importance of determining osteoprotegerin concentration as a possible prognostic and diagnostic marker in other cancers. Such studies were conducted by Mizutani et al. [40], who were the first to assess the prognostic significance of determining the serum osteoprotegerin levels in patients with bladder cancer. Their research showed that the serum OPG concentration correlated with the severity of the disease in patients with bladder cancer, and the increased serum OPG levels were associated with early relapse. The correlation between the concentration of the measured parameter and the postoperative prognosis demonstrated by the authors indicates that the determination of OPG serum concentration may be useful as a prognostic marker in patients with bladder cancer, and also this determination may be helpful in the selection of patients for intensive surgical or chemotherapeutic treatment [40]. Eaton et al. [41] evaluated OPG levels in untreated prostate cancer patients with advanced prostate cancer compared to patients with organ-confined disease and in treated patients receiving androgen ablation. According to the authors, OPG might be a potential new marker that is elevated in the serum of patients with advanced prostate cancer and may be an indicator of early disease progression [41]. Lipton et al. [42] evaluated the OPG concentration in the serum of patients with selected haematological and solid tumours. The analysis showed increased serum OPG concentration in patients with colorectal cancer and pancreatic cancer compared to healthy patients. However, the analysis of the concentration of the studied parameter, taking into account the tumour metastasis site, showed a significant increase in OPG in the serum only in patients with liver and soft tissue metastases. The authors also showed that OPG levels were elevated in the serum of patients with Hodgkin’s disease and non-Hodgkin’s lymphoma, and decreased in the serum of patients with multiple myeloma. In their opinion, despite the fact that some patients with malignancy have significant elevations of circulating OPG, these concentrations do not approach the level that would be expected to suppress osteoclast function [42]. Mercatali et al. [43], on the other hand, evaluated the usefulness of circulating OPG and RANK-L to detect bone metastases. The researchers highlighted a potentially important role of circulating OPG in the diagnosis of bone metastases [43]. Also, Elfar et al. [44] assessed the role of osteoprotegerin and NF-κB ligand receptor activator as parameters helpful in detecting bone metastases in breast cancer. The authors showed that OPG serum levels are significantly reduced in patients with breast cancer and bone metastases compared to those in patients with nonmetastatic breast cancer. OPG levels correlated negatively with CA 15-3 levels. Interestingly, as studies have shown, OPG was produced not only in the bone microenvironment by osteoblasts, but also by breast cancer cells. According to the authors, OPG plays a potential role in breast cancer by being able to block the induction of apoptosis [44]. Similar studies were also conducted by Martinetti et al. [45], who showed that osteoprotegerin (OPG) and osteopontin (OPN) appear to be useful predictors of the outcome of skeletal disease and elevated OPN values may be associated with short survival in advanced breast cancer patients.

## 5. Conclusions

To sum up, based on the research we concluded that:

1. An important role in the pathogenesis of ovarian cancer is played by receptor-mediated apoptosis disorders involving soluble particles sTRAIL and osteoeprotegerine, which are not only one of the important immunological mechanisms of immunosuppression associated with ovarian cancer, but may also promote the development of this tumour.
2. The observed changes in the sTRAIL/OPG system concentration in the serum of women with ovarian cancer are related to the histological type of ovarian cancer and the degree of histological differentiation of this cancer, which may be used in the development of new immunotherapy methods.
3. The determination of sTRAIL and OPG in combination with other markers may be useful in the future in the diagnosis of ovarian cancer, but that is requires further research.

## Figures and Tables

**Table 1:** Sensitivity, specificity, positive predictive value (PPV), and negative predictive value (NPV) determination of TRAIL and CA 125 antigen.

Marker	Sensitivity	Specificity	PPV	NPV
TRAIL	100%	100%	0%	0%
CA 125	87.62%	87.09%	12.90%	12.37%

PPV—positive predictive value; NPV—negative predictive value.

**Table 2:** Sensitivity, specificity, PPV, and NPV determination of OPG and CA 125 antigen concentration.

Marker	Sensitivity	Specificity	PPV	NPV
OPG	95.88%	90.32%	9.67%	4.12%
CA 125	87.62%	87.09%	12.90%	12.37%

PPV—positive predictive value NPV—negative predictive value.
